# Galician consensus on management of cardiotoxicity in breast cancer: risk factors, prevention, and early intervention

**DOI:** 10.1007/s12094-017-1648-8

**Published:** 2017-03-24

**Authors:** J. F. Cueva, S. Antolín, L. Calvo, I. Fernández, M. Ramos, L. de Paz, J. G. Mata, R. López, M. Constenla, E. Pérez, A. González, M. L. Pellón, S. Varela, T. López

**Affiliations:** 1 Oncology Department, Complejo Hospitalario Universitario Santiago de Compostela, Santiago de Compostela, Spain; 2 Oncology Department, Complejo Hospitalario Universitario A Coruña, A Coruña, Spain; 3 Oncology Department, Complejo Hospitalario Universitario Vigo, Vigo, Spain; 4 Oncology Department, Centro Oncológico Galicia, A Coruña, Spain; 5 Oncology Department, Complejo Hospitalario Universitario Ferrol, Ferrol, Spain; 6 Oncology Department, Complejo Hospitalario Universitario Ourense, Orense, Spain; 7 Oncology Department, Complejo Hospitalario Universitario Pontevedra, Pontevedra, Spain; 8 Oncology Department, Complejo Hospitalario Universitario Lugo, Lugo, Spain; 9 Cardiology Department, Hospital Universitario La Paz, Madrid, Spain

**Keywords:** Cardiotoxicity, Breast cancer, Anticancer therapies, Cardiovascular risk factors

## Abstract

This Galician consensus statement is a joint oncologists/cardiologists initiative indented to establish basic recommendations on how to prevent and to manage the cardiotoxicity in breast cancer with the aim of ensuring an optimal cardiovascular care of these patients. A clinical screening of the patients before treatment is recommended to stratify them into a determined risk group based on their intrinsic cardiovascular risk factors and those extrinsic arose from breast cancer therapy, thereby providing individualized preventive and monitoring measures. Suitable initial and ongoing assessments for patients with low and moderate/high risk and planned treatment with anthracyclines and trastuzumab are given; also, measures aimed at preventing and correcting any modifiable risk factor are pointed out .

## Introduction and methodology of Consensus Meeting

Improved survival of cancer patients in recent decades, together with the toxicities of antitumor drugs, the increased population of patients over 65 years (with comorbidities), and an inadequate knowledge and management of risk factors, has led to an increase in cardiovascular morbidity in cancer patients [1]. Chemotherapy is a cardiovascular risk factor, even more with radiotherapy, increasing by 30% the incidence of events compared to the general population [2]. Considering the high frequency of cardiovascular risk factors and the presence of preexisting cardiovascular disease, it is of key importance to optimize and standardize the management of these patients, in a multidisciplinary approach that we could call Cardio-Oncology [1]. Breast cancer was chosen for this consensus because of its incidence and high survival rates, which grants greater importance to prevention, control and monitoring of toxicity, and because of the routine use of radiotherapy and cardiotoxic drugs. With this purpose, the following methodology was applied: first, one cardiologist presentation; followed by *two workshops* (medical oncologists), with discussion and conclusions of each one; followed by sharing perspectives on key issues and consensus; and finally, manuscript drafting and review.

## Objectives

- To establish the clinical cardiovascular risk factors and those intrinsic to treatment in breast cancer patients.
- To establish the basis for prevention of cardiotoxicity related to anticancer treatments for breast cancer.
- To establish multidisciplinary cardio-oncological bases for early intervention in the management of cardiotoxicity.
- Finally, to establish basic recommendations agreed by consensus for prevention, initial management, and referral.

## The cardiologist’s viewpoint

Cardiac dysfunction related to cancer treatment has been defined as a decrease in left ventricular ejection fraction (LVEF) by ultrasound greater than 10% (from baseline) and with an absolute value less than 53%, confirmed by a repeat examination at 2–3 weeks [3]. LVEF between 53 and 73% is considered normal. At least two types of mechanisms of cardiotoxicity are recognized, according to the presence or not of structural anomalies and their reversibility [4]. In type I (adriamycin model), myocardial cell necrosis/apoptosis occurs in a dose-dependent manner, causing permanent damage (visible on biopsy), and for which early diagnosis, prevention, and treatment are essential. In type II (trastuzumab model), cellular dysfunction without apparent structural damage occurs, due to blockade of cellular survival pathways associated with HER2 and activated by stress, there appears to be no cumulative effect, and the damage is reversible in the majority of cases with drug discontinuation [5]; and for its prevention, the knowledge of risk factors and monitoring of treatment are very important. It should be noted that cardiotoxicity is potentiated by the combination of anthracyclines and trastuzumab [6]. Nevertheless, the finding on cardiac magnetic resonance imaging (MRI) of scars in patients with type II toxicity, as well as the improvement in cardiac function with adequate early treatment in some type I cases [7], indicates that this classification may not be so strict. Moreover, while anthracyclines and anti-HER2 agents make up the two large groups of cardiotoxic drugs, other cytotoxic drugs, other monoclonal antibodies, and certain tyrosine-kinase inhibitors and antiangiogenic drugs may also be cardiotoxic through different mechanisms.

Cardiac damage initially occurs in a molecular phase, followed by cellular damage, asymptomatic dysfunction, and finally symptomatic clinical dysfunction. Our diagnostic intervention is currently based on monitoring LVEF by ultrasound, multigated acquisition (MUGA) scan or MRI, considering <53% as abnormal. Although the reference technique for quantification of LVEF is cardiac MRI, ultrasound offers the advantages of its availability, low cost, lack of radiation, and overview of cardiac function. However, 2D ultrasound depends on the quality of the image and the expertise of the operator. Furthermore, it has a reported variability of about 10%, similar to the value used for diagnosis of cardiotoxicity. New non-enhanced 3D imaging techniques reduce this variability and are considered the ideal method for monitoring patients treated with cardiotoxic drugs [8].

However, the measurement of LVEF is able to diagnose and quantify but does not predict the development of cardiotoxicity. We need other parameters to detect early changes predictive of late morbidity and mortality. The cardiac muscle is formed by three layers of myocardial fibers with different orientations, and systolic function of the left ventricle is the sum of longitudinal contraction, circumferential shortening, and radial thickening. Measurement of LVEF only evaluates radial function [9, 10]. New imaging techniques can provide information in earlier stages. The most widely used are those quantifying myocardial deformation, and the most studied parameter is deformation of longitudinal fibers or global longitudinal strain (GLS). Its normal value in healthy subjects is −19.7%, with less than 4% of variability [11–13]. A review (*n* = 384) showed that GLS changes are frequent during treatment with anthracyclines and occur earlier than LVEF changes [14]. In addition, cardiac biomarkers (basically troponins) are also a useful tool in monitoring of cardiotoxic treatments. They are simple to use and have very low variability between determinations [15, 16]. Thus, a 10–15% early reduction in GLS combined with a rise in troponins has been shown as a good predictor of clinical events or ventricular dysfunction. If GLS and troponins are normal at 6 months after completing treatment, the risk of dysfunction is low, which shows that the combination of these parameters has a high predictive value [14].

Despite the above, only 30–35% of patients receiving cardiotoxic therapies who present with asymptomatic left ventricular dysfunction receive beta-blockers (BB) or angiotensin-converting-enzyme inhibitors (ACEIs)/angiotensin II receptor antagonists (ARA II); and only about 40% receive a cardiological consultation [17]. Management of these patients should be performed according to current cardiological guidelines, such as the ACCF/AHA guideline [18]. When these patients are referred for cardiology consultation, prescription of ACEIs and BBs increases along with survival [19]. It is important to note that when dysfunction develops, the probability of complete recovery is reduced despite optimal treatment [7]. But it should also be stressed that early intervention can stop damage progression and improve cardiac function. It has been estimated that 40–55% of patients can normalize their LVEF even after having developed clinical cardiotoxicity [20]. The efficacy of ACEIs (enalapril type) as first-line treatment has been shown in breast cancer patients after anthracycline-based chemotherapy, by improving cardiac function [21]. BBs (carvedilol or bisoprolol type) are the second essential drugs, and combined carvedilol and enalapril in low doses has demonstrated its utility, too [7]. In this way, ACEIs/ARA II and/or BBs have already shown that enhances recovery of LVEF and improves cardiovascular prognosis [22]. The key is the earliness of the start of treatment. So, delays greater than 6 months considerably reduce the chances of success. Once LVEF falls below 53%, even in the absence of symptoms, it is recommended to start early treatment with progressively higher doses according to clinical tolerance [8]. In addition, early intervention to promote cardio-healthy lifestyle habits and to control existing cardiovascular risk factors is also essential. The goals are to maintain a body mass index (BMI) between 18 and 25, not to smoke, do exercise, maintain low-density lipoprotein (LDL) cholesterol levels below 100 and glycated hemoglobin (HbA1c) levels below 7%, and to control arterial hypertension, if appropriate [23]. Finally, depending on its course, reversibility can now be classified into four groups: reversible, if LVEF improves more than 10% with a difference from baseline ≤5%; partially reversible, if LVEF improves but differences persist with baseline >5%; irreversible, if LVEF improves less than 10% and maintains differences with baseline >5%; and indeterminate if no follow-up is performed).

**Fig. 1:**
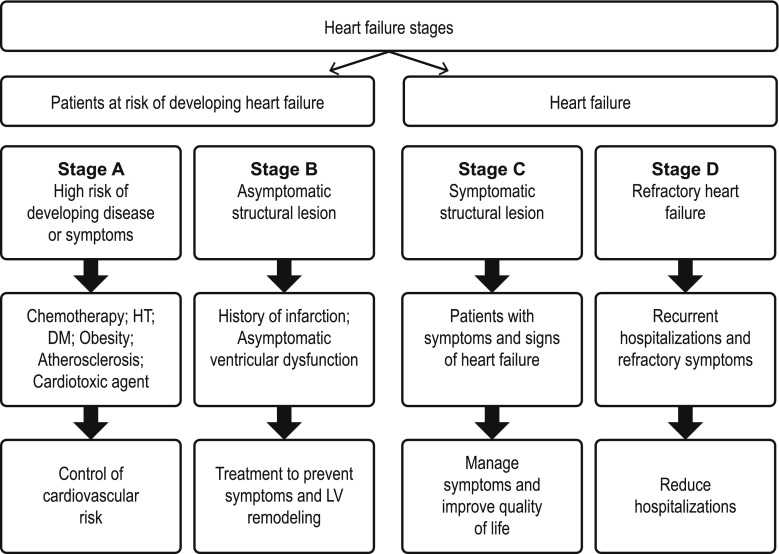
Adapted ACCF/AHA 2013 guideline for the management of heart failure. *HT* hypertension, *LV* left ventricular

In summary, early prevention of cardiotoxic events in patients undergoing cancer treatment is essential. Therefore, and to minimize the cardiotoxicity of treatment as far as possible, risk should be evaluated individually, identifying and controlling cardiovascular risk factors, and thereby permitting early diagnosis and treatment. The objective initially will be to detect which patients are at risk of developing stage B heart failure (Fig. 1) and to intervene actively both in lifestyle modification and control of cardiovascular risk factors.

## Workshop 1: Cardiological risk factors of patients undergoing breast cancer treatment

- Age: extreme ages, very young or ≥65–70 years, are a risk factor. In a retrospective study it was observed that the risk of suffering heart failure (HF) due to anthracyclines increased with age [24]. Other retrospective study showed a 2.25-fold higher risk of HF after a total adriamycin dose of 400 mg/m^2^ in patients older versus younger than 65 years [25]. Similarly, age >65 years has been related to an increased risk (HR 2.08) of cardiotoxicity with trastuzumab in a retrospective analysis [26].
- Gender: being a woman, especially postmenopausal, is a risk factor; although it is controversial in cancer treatment because the literature offers contradictory results. In long-term follow-up studies of childhood cancer survivors treated with anthracyclines, greater cardiotoxicity was observed in women [27]. However, in a study of adult patients with lymphoma, male gender was correlated to greater cardiotoxicity than female [28].
- Smoking: the relationship between smoking and heart disease is widely documented.
- Sedentarism: it is also an important cardiovascular risk factor, considered as less than 150 min per week of moderate exercise.
- Obesity: the presence of a BMI ≥25 is also considered a risk factor. Weight ≥70 kg has been identified as predictive factor for cardiotoxicity in advanced breast cancer treated with anthracyclines [29]. A BMI ≥27 has also been correlated to an increased incidence of cardiac dysfunction in patients with adjuvant epirubicin-based chemotherapy versus a BMI <27 (1.8 vs 0.9%) [30]. These results could be explained at least in part by the fact that obese patients received higher doses of anthracyclines if calculated by their real body surface.
- Diabetes mellitus (DM): it is also a well-documented cardiovascular risk factor.
- Dyslipidemia: total cholesterol/LDL-cholesterol ratio is another well documented risk factor.
- Arterial hypertension (HT): control of blood pressure reduces the HF risk by over 50% [31, 32]. It should be performed according clinical guidelines [18]. A meta-analysis has suggested that diuretics, ACEIs and ARA-II are the most effective drugs [32].
- Preexisting cardiovascular disease: It is common that at time of diagnosis, breast cancer patients already have risk of developing a cardiovascular disease, which will be increased by the effect of treatment, in what is known as the “multiple-hit” hypothesis [33]. Furthermore, it has been reported that a previous cardiovascular disease increases the risk of developing HF after anthracyclines, with a HR for myocardial infarction or atherosclerosis of 2.21 and of 1.53 for any other previous cardiovascular disease [34].

Based on these factors we finally established two large risk groups:

- Low risk: asymptomatic patients without risk factors.
- Moderate/high risk, with >5% of cardiac events at 10 years: presence of ≥2 factors listed above.

## Workshop 2: cardiotoxicity risk factors related to breast cancer treatment

- Local treatment: chest wall/mediastinal irradiation: Radiotherapy affects the heart at various levels: valvular, vascular, pericardium, and myocardium, especially if 30 Gy are exceeded [1, 35] and/or the internal mammary chain is irradiated. The incidence of cardiotoxicity related to irradiation is estimated to be 10–30% in the 5–10 years following treatment [36]. Moreover, damage caused by anthracyclines is more common in previously radiated patients [37]. A randomized study with adriamycin with or without radiotherapy showed an increase in cardiac events with doses over 450 mg/m^2^ and radiotherapy [38]. However, with modern planning and techniques, irradiation of cardiac structures can be reduced to a minimum.
- Cytotoxic systemic treatment: anthracyclines: Anthracyclines have the highest rate of acute (myopericarditis, arrhythmias) and chronic cardiac toxicity. They associate with a risk of irreversible progressive cardiomyopathy of 3–26%. In a meta-analysis (22,815 patients), adriamycin were associated with a 6 and 18% risk of clinical and subclinical cardiotoxicity, respectively; a 10% rate of events; and a 0.4% of deaths of cardiac origin [39]. Their risk is clearly related to the cumulative dose. Thus, HF risk with a standard regimen (bolus dose every 3 weeks) and a cumulative dose < 300 mg/m^2^ is about 2%, although this risk begins to significantly increase from 300 mg/m^2^ [6]. Their cardiac toxicity also depends on the frequency of administration and of rate of infusion. Thus, 6 h or longer infusions or weekly schedules show less cardiotoxicity than standard boluses, by avoiding high plasma peaks [37]. And, of course, their toxicity is increased in presence of radiotherapy and other cardiovascular risk factors, including the use of other cardiotoxic drugs such as taxanes or trastuzumab. In addition to adriamycin, there are available other anthracyclines. Epirubicin is an epimer of adriamycin apparently less cardiotoxic, at least when it is used in equivalent myelosuppressive doses of up to 700–800 mg/m^2^. Its incidence of cardiomyopathy is of 1–3%. A study showed a 1.9% risk with doses of up to 800 mg/m^2^, increasing to 4.3% with 900 mg/m^2^ [40]. These data are similar to those reported with adriamycin, so probably the difference is simply that cardiotoxic doses are not usually reached with epirubicin. There are also studies in which concomitant trastuzumab–epirubicin (FEC) is highly effective in HER2+ breast cancer treatment, without relevant cardiotoxicity at the doses used [41].
*Liposomal anthracycline formulations* are clearly less cardiotoxic as has been demonstrated in at least three randomized studies. They were developed to improve the therapeutic index of conventional anthracyclines. Liposomal encapsulation prevents its extravasation into capillaries of cardiac muscle, facilitating its passage into immature vascular systems (tumor vessels), reduces its volume of distribution and diffusion, and also reduces plasma peaks. So, maintaining its antitumor activity toxicity is generally lower in healthy tissues [42]. There are two liposomal-encapsulated formulations of adriamycin, pegylated (PLA, CAELYX^®^) and non-pegylated (NPLA, MYOCET^®^). The latter has a reduced myocardial uptake and rapid clearance, preventing the occurrence of toxic peaks. Both were at least as effective as conventional adriamycin in first-line treatment of advanced breast cancer. Thus, a randomized study showed equivalent efficacy and reduced cardiotoxicity (4 vs 19%) of PLA versus adriamycin, with increased hand-foot syndrome (HFS) [43]. Two randomized trials showed that NPLA is significantly less cardiotoxic than conventional adriamycin (13 vs 29% in one trial, and 6 vs 21% in the other trial), with comparable efficacy [44, 45], with a trend to reduced gastrointestinal toxicity and neutropenia, and negligible HFS [46]. A pooled analysis from these two trials has suggested that NPLA could be even more effective than conventional adriamycin, in terms of response rate (31 vs 11%) and progression-free survival (PFS) (4.2 vs 2.1 months) [42]. Finally, a meta-analysis showed significantly lower rates of both clinical (RR 0.20) and subclinical heart failure (RR 0.38) with NPLA compared with adriamycin [46]. Based on the above, liposomal anthracyclines are a reasonable alternative in patients with anthracycline therapy indication and increased cardiac risk. This would include having received adjuvant anthracycline therapy at dangerous cumulative doses.
- Cytotoxic systemic treatment: other cytotoxic agents: *Cyclophosphamide*: Cyclophosphamide acute cardiotoxicity (myocardiopericarditis) is generally related to high doses and usually resolve without sequelae after discontinuing the drug. *Taxanes*: Paclitaxel has been related to disturbance in cardiac rhythm, mainly episodes of acute symptomatic bradycardia, and when used in combination with adriamycin it has been shown to increase the cardiotoxicity of the latter in a sequence-dependent manner, so the administration should be paclitaxel first, followed by adriamycin [47]. *Other cytotoxic agents*: in addition to the vascular toxicity (ischemia) of the fluoropyrimidines, different incidences of cardiomyopathy have been reported with ifosfamide, cisplatin, and vincristine.
- Endocrine systemic treatment: estrogens have beneficial effects on lipids in postmenopausal women, so estrogen cessation could increase cardiovascular risk. However, the cardiac risk of antiestrogen treatment is insufficiently studied. In advanced disease, it appears that ischemic events (1–4%) and arrhythmias (4–7%) are the most predominant [48]. *Tamoxifen* has been related to venous thrombotic events, and it does not appear to exert a clear cardioprotective effect despite reducing cholesterol levels. *Aromatase inhibitors* in general increase cholesterol levels, at least when compared to tamoxifen, and some studies have reported more cardiac events (versus tamoxifen). Nevertheless, the data in the literature are few, discordant, and inconsistent.
- Anti-HER2 systemic treatment: *Trastuzumab*: Trastuzumab, a monoclonal antibody against HER2, is known to be cardiotoxic, especially in combination with anthracyclines. In a pivotal trial in advanced breast cancer, the incidence of HF was 16% in the Adriamycin (AC) + trastuzumab arm versus 3% in the AC arm [49]. As adjuvant therapy, the incidence of cardiac events was also higher with anthracyclines plus trastuzumab (4.1%) versus anthracyclines (0.8%) [50]. A meta-analysis of 11,882 patients showed that HF risk significantly increased if the patients also received anthracyclines (RR 4.19 in initial, 4.75 in advanced disease), but not in those who did not [51]. In the Breast Cancer International Research Group 006 study to evaluate adjuvant trastuzumab, clinical HF rate was five times lower (0.38 vs 1.96%) when trastuzumab was used without anthracyclines (docetaxel–carboplatin) [52]. Other adjuvant therapy study with trastuzumab (*n* = 1664) showed some predictors of cardiotoxicity: age, previous cardiovascular conditions, and the characteristics of the treatment received [53]. Although trastuzumab-induced cardiotoxicity (type II) has not traditionally been considered as cumulative, in the HERA study an increased risk of discontinuing treatment due a cardiac cause was reported in the 2-year trastuzumab arm versus the 1-year arm [54]. However, after a median follow-up of 8 years, the incidence of cardiac events was very low, without differences between the 1- and 2-year arms (0.8%) [55]. Moreover, in the Finn-HER study, with only 9 weeks of trastuzumab, cardiotoxicity was minimal [56]. *Other anti*-*HER2 drugs*: with different mechanisms of action, pertuzumab, trastuzumab-emtansine (T-DM1), and lapatinib appear to be less cardiotoxic. In advanced disease, the incidence of asymptomatic cardiac events with lapatinib has been reported to be of 1.4%; as for pertuzumab combined with trastuzumab, a rate of ventricular dysfunction of 8%, mainly asymptomatic [57] was observed. In the phase III pivotal study with T-DM1, the reported incidence was also very low [58].

## Discussion, general recommendations, and conclusions

It is essential to research on cardiotoxicity of anticancer agents, primary prevention, and early detection of injury, as well as monitoring and treatment of early heart damage [59]. In addition to survival, the goal of oncologists should be to reduce toxicities, especially late toxicities and particularly cardiotoxicity. As we have seen, we must to optimize the strategies to reduce the risk of cardiovascular events. Once toxicity has occurred, even if asymptomatic, cardiological assessment is recommended, and a close structured collaboration between cardiologists and oncologists should be required [39].

**Fig. 2:**
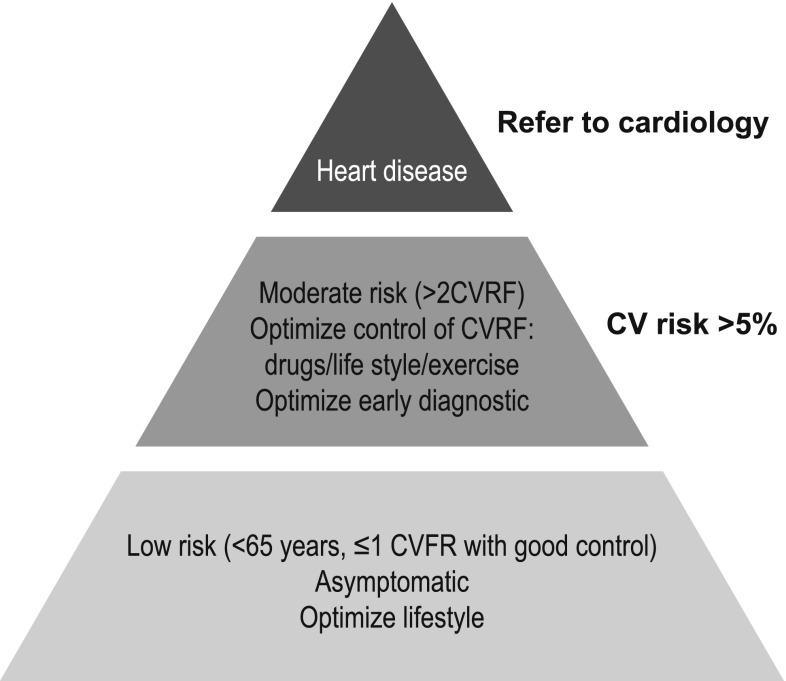
Pyramid for management of cardiotoxicity risk. *CVRF* cardiovascular risk factors, *CV* cardiovascular

It is possible to calculate, prior to initiating treatment, an overall risk score of developing cardiotoxicity. This risk would be the sum of the following points (maximum, 14): medication risk score (4 points for anthracyclines and trastuzumab, among others); plus one point for each cardiovascular risk factor (women, <15 years or over 65, history of cardiomyopathy or congestive heart failure, ischemic heart disease, arrhythmias, corrected QT >500 ms, hypertension, diabetes), previous use of anthracyclines and/or thoracic radiotherapy [1] (Table 1). In this way, a pyramid can be constructed according to the individual risk of each patient. At the base would be patients with low risk (<65 years, ≤1 risk factor and good control) who are asymptomatic and for whom the intervention would be to optimize lifestyle and control the use of cardiotoxic agents. At the top would be patients with symptomatic heart disease, with moderate-severe ventricular dysfunction, in whom the use of cardiotoxic agents should be avoided. And between both would be patients with moderate risk (>2 risk factors), asymptomatic but with a risk of heart disease >5%. In these cases, added to optimizing lifestyle and controlling risk factors, the intervention should be aimed at adjusting as much as possible the use of anthracyclines or assessing the need of liposomal formulations; and in any case, an adequate monitoring of potentially cardiotoxic treatments to perform early diagnosis of injury (Fig. 2) should be established.

**Table 1:** Cardiotoxicity risk assessment

Risk of cardiotoxicity related to cancer treatment	Criteria for high CV risk of developing cardiotoxicity
High risk Anthracyclines, cyclophosphamide, ifosfamide, trastuzumab, and clofarabine Intermediate risk Docetaxel, pertuzumab, sunitinib, and sorafenib	History of: Cardiomyopathy or heart failure Ischemic heart disease Arrhythmias under treatment QTc >500 ms
Low risk Bevacizumab, dasatinib, imatinib, lapatinib, etoposide, rituximab, and thalidomide	HT Diabetes Previous use of anthracyclines Mediastinal radiotherapy Female gender <15 years or >65 years

*CV* cardiovascular, *HT* hypertension, *QTc* corrected QT interval

**Fig. 3:**
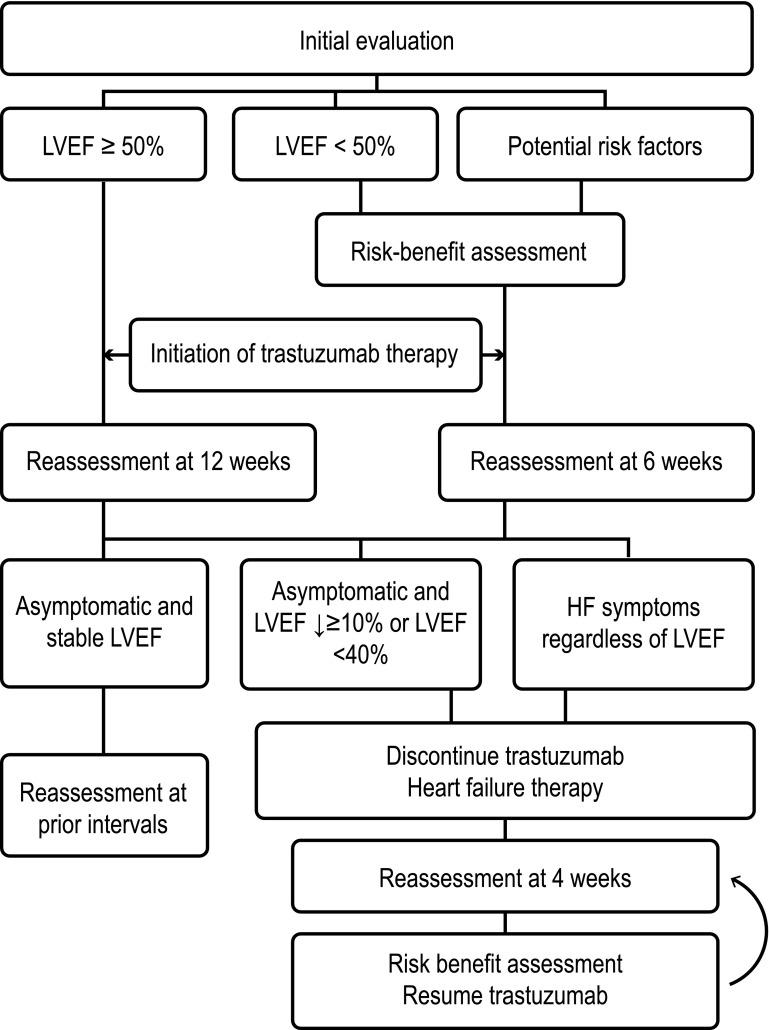
Sample algorithm for trastuzumab monitoring. (From *The Oncologist* [61], with permission). *HF* heart failure, *LVEF* left ventricular ejection fraction

There are several algorithms published for monitoring patients receiving treatment with anthracyclines [60], trastuzumab [61] (Fig. 3); and radiotherapy [62]. It is generally recommended to perform a baseline LVEF assessment in all patients who start a cardiotoxic treatment, with the frequency of subsequent monitoring depending on the calculated risk. With anthracyclines, it is generally recommended to repeat the assessment at the end of treatment and at 6 months, and if the dose exceeds 300 mg/m^2^, before each cycle. For trastuzumab, it is recommended that assessments are performed every 3 months during active treatment and at 6 months after the end of treatment [3]. However, this regimen may be difficult to apply in clinical practice, so it is advisable for oncology and cardiology teams of each center to establish a plan of minimum requirements including an early referral path to cardiologist (Table 2). Diagnosis of cardiotoxicity is currently made by measurement of LVEF, optimally by 3D echocardiography. In case of 2D LVEF, use Simpson’s method ± contrast or MRI when in doubt or before making changes in treatment due to cardiotoxicity. The ideal strategy to anticipate risks seems to be changes related to GLS ± alteration of troponins so that if both are abnormal, the positive predictive values is 94% (diagnosis), and if both are normal, the negative predictive value is 97%.

**Table 2:** Monitoring and referral to cardiology

Cardiotoxicity risk	Monitoring
High risk drugs + established heart disease and LVEF <40%	Carefully assess indication for cardiotoxic agents. Individualize, support from cardiology
High/moderate risk drugs + CVRF or LVEF >40%	*Baseline*: ECG; blood test (HbA1c, lipids and troponin?); LVEF measurement (2D/3D US ± GLS) *During treatment*: control CVRF, troponin in each cycle?; assess BB, ACEIs, and statins *End of treatment*: troponin?; ECG, LVEF measurement (2D/3D US ± GLS) *Follow*-*up*: measure LVEF at 6 months from end of treatment (2D/3D US ± GLS), and every 3–4 years
High/moderate risk drugs + asymptomatic without CVRF	*Baseline*: ECG; blood test (HbA1c, lipids, and troponin?) *End of treatment:* troponin?; ECG, LVEF measurement (2D/3D US ± GLS) *Follow*-*up*: according to symptoms
Criteria for referral to cardiology Patients with high or intermediate risk of cardiotoxicity, to optimize medical treatment: BB, ACEIs, statins Previous treatment with adriamycin ≥300 mg/m^2^ Mediastinal irradiation ≥30 Gy Previous heart disease (cardiomyopathy, heart failure, arrhythmias or ischemic heart disease), if not followed up in cardiology Poorly controlled CVRF with treatment: HT, diabetes, dyslipidemia, smoking Alterations at baseline: ECG, troponin or LVEF measurement Alterations during follow-up: LVEF decrease >10% or LVEF <53% Abnormal GLS (>−19%) or decrease >15% Positive troponin I Chest pain, dyspnea on exertion, syncope, arrhythmias HT refractory to conventional treatment	

*ACEIs* angiotensin converting enzyme inhibitors, *BB* beta-blockers, *CVRF* cardiovascular risk factors, *ECG* electrocardiogram, *GLS* global longitudinal strain, *Hb* hemoglobin, *HT* hypertension, *LVEF* left ventricular ejection fraction

It is known that prevention strategies including cardio-healthy lifestyle habits (healthy diet, regular exercise, control of BMI or abdominal fat, avoidance of smoking and alcohol), as well as control of clinical risk factors (hypertension, dyslipidemia, or hyperglycemia), are associated with a lower incidence of cardiovascular disease [63–65]. In this regard, statins in 628 patients with breast cancer receiving anthracyclines reduced the incidence of HF [66]. Moreover, atorvastatin has shown cardioprotection in patients treated with anthracyclines [67]. In a retrospective study on patients with normal LVEF prior to treatment with anthracyclines and trastuzumab, 106 patients have received beta-blockers (BBs) during chemotherapy, which was associated with a significant reduction in HF (HR 0.2) [68]. In a prospective study, enalapril reduced the incidence of ventricular dysfunction compared with placebo in patients with troponin elevation after anthracyclines [69]. The OVERCOME study involved 90 hematological tumor patients and showed that after 6 months of treatment with enalapril plus carvedilol the incidence of cardiac events significantly decreased [70]. On the other hand, it has been reported that not all BBs offer the same protection against cardiotoxicity. Non-selective BB such as propranolol could even potentiate toxicity [71], while carvedilol and nebivolol have shown to be protective [72–75].

Based on the information reviewed above, it is possible to establish protective strategies for individual risk. The risk of cytotoxic agents can be prevented by standardized protection protocols, optimizing their use (schedule, dosage), and including the option of less cardiotoxic agents use. To prevent the risk of thoracic irradiation, planning should be optimized and doses reduced as much as possible (<30 Gy in total, <2 Gy daily). Regarding individual risk factors, those that are modifiable include hypertension, diabetes, dyslipidemia, smoking, sedentarism, and treatment of heart diseases in general. Aerobic cardiovascular training is fundamental to metabolic control, and a very effective method to improve quality of life and capacity of exercise in cancer patients. Furthermore, it has been published that regular physical activity (3–5 h of moderate exercise a week, equivalent to walking 30 min at least 5 days a week) reduces cancer mortality by 30–50% in patients treated with curative intent [76]. So, it is possible to establish general recommendations about lifestyle habits (Table 3). However, age, female sex, and genetic predisposition are not modifiable factors.

**Table 3:** Healthy lifestyle recommendations

No smoking
Limit salt and alcohol consumption (1–2 glasses of red wine a day)
Practice exercise: walk 30 min at least 5 days a week
Adopt a Mediterranean diet: 5–6% saturated fat; 26–27% fat; 15–18% proteins; 55–59% carbohydrates
Check weight periodically and consult in case of sudden increases or presence of edema
Control cholesterol, glucose, and blood pressure (<140/85)
Consult in case of shortness of breath or chest pain with exercise, palpitations or blackouts

The final objective should be to evaluate individual risk of cardiotoxicity in each patient, in order to, on the one hand, apply preventive measures and optimize management and control of modifiable risk factors; and on the other hand, avoid the use of cardiotoxic drugs based on the risk/benefit assessment or, as alternative, use drugs with less cardiotoxic profile. Cardioprotective drugs like dexrazoxane (iron chelator), although they have been shown to reduce cardiotoxic events [77], are not recommended by the American Society of Clinical Oncology due to the risk of bone marrow suppression and second cancers, and especially because of doubts about a reduction in the efficacy of the cytotoxic agent [78]. Therefore, the best option, if anthracyclines are indispensable, is the use of agents with a reduced cardiac toxicity profile, such as liposomal anthracyclines [79].

### Key messages [80]

- Cardiovascular disease is the most common cause of mortality in patients who have survived a cancer. A multidisciplinary approach is essential in order not to compromise the prognosis of these patients.
- Patients treated with potentially cardiotoxic drugs are encompassed in stage A of heart failure.
- Clinical screening is required before treating patients with breast cancer to stratify them according to the calculated risk and to apply adequate preventive and monitoring measures.
- Early and optimal control of cardiovascular risk factors is essential to improve the diagnosis. Preliminary studies support primary preventions with ACEIs (enalapril), BBs (carvedilol, nebivolol), and/or statins (atorvastatin).
- It is necessary to optimize the indications of cardiotoxic agents and to apply preventive measures from the beginning, which may include the use of cardioprotective drugs (in very select cases) or liposomal formulations of anthracyclines.
- The current recommendation is to monitor LVEF with 3D echocardiography (2D if not available), together with contrast administration in case of a nonoptimal window. If the assessment is still suboptimal, or before modifying treatment for ventricular dysfunction, it is recommended to confirm LVEF by cardiac magnetic resonance imaging. The use of GLS and high-sensitivity troponins allows early diagnosis of subclinical myocardial injury.
- Early treatment of myocardial injury is vital to improve the cardiovascular prognosis and the quality of life of survivors. The probability of recovering LVEF depends primarily on the earliness of treatment.
- Multidisciplinary collaboration by the cardio-oncology team ensures optimal cardiological care of oncological patients and increases the safety of treatments.

## Consensus recommendations

Individualized initial and ongoing assessment according to risk factors:

- *Established clinical risk group*:
  - Low risk: asymptomatic patient without risk factors.
  - Moderate/high risk: presence of ≥2 risk factors listed in *Workshop 1*.
- *Low risk assessment*:
  - *Planned treatment with anthracyclines*, clinical assessment, baseline ECG and blood tests (including fractionated lipid profile and HbA1c), and LVEF assessment at end of treatment.
  - *Planned treatment with trastuzumab* in addition to the previous points, a baseline and some intermediate LVEF assessment during treatment is recommended.
- *Moderate/high risk assessment*:
  - *planned treatment with anthracyclines*, clinical assessment, baseline ECG and blood tests (including fractionated lipid profile and HbA1c), and LVEF assessment at baseline and the end of treatment.
  - *Planned treatment with trastuzumab* in addition to the above, it is recommended with low consensus level, to adjust the intermediate LVEF monitoring (every 3–6 months) to existing risk factors and the decreases observed in previous LVEF measurements.
  - Among attendees there was the idea that in addition to the above, monitoring of serum levels of high-sensitivity troponins before each cycle (in centers where this is available) could optimize follow-up and improve both prevention and early therapeutic intervention, but a consensus was *NOT* reached for its generalized use. If troponin monitoring is used and an elevation is observed, an echocardiogram should be performed and consider, depending on each case, to start treatment with BB or ACEIs in order to prevent long-term ventricular dysfunction and continue cancer treatment.

Individualized and dynamic initial and ongoing intervention:

- prevention and correction of modifiable risk factors (Table 3): this requires recommendations not only involving each patient individually, but all healthcare personnel intervening in their treatment (nursing, other specialists, primary care), as well as patient associations, the media, and in general society as a whole.
- Optimize cardiovascular treatment of these patients. If drugs for blood pressure control are needed to opt for ACEIs (e.g., enalapril) and/or BBs (e.g., carvedilol, nebivolol) and discontinue calcium antagonists, especially verapamil or diltiazem (negative intropes). With respect to the use of statins, there is no clear evidence to start therapy with normal cholesterol levels. The key is a strict control of LDL levels and to start statins (e.g., atorvastatin) with LDL >100 mg/dL.

### Referral to cardiologist

It is recommended to provide rapid communication channels between specialists and to consult cardiologist for treatment changes. Early treatment of myocardial injury (with ACEIs and/or BBs) is vital to improve the cardiovascular prognosis. Moreover, cardiologist intervention should be mandatory in case of an abnormal ECG or baseline measurement of LVEF, a significant decrease in LVEF during monitoring, a sustained and confirmed poor blood pressure control (>140/85), and in the presence of cardiac alarm symptoms or signs such as arrhythmias, syncope, chest pain, dyspnea, edema, etc.
